# Applying Technology Acceptance Model in Online Entrepreneurship Education for New Entrepreneurs

**DOI:** 10.3389/fpsyg.2021.713239

**Published:** 2021-10-12

**Authors:** Yawen Su, Moyan Li

**Affiliations:** ^1^The Education University of Hong Kong, Tai Po, Hong Kong, SAR China; ^2^School of Humanistic and Social Sciences, Shantou Polytechnic, Shantou, China

**Keywords:** technology acceptance model, online education, entrepreneur, innovation and entrepreneurship, self-efficacy and perceived usefulness

## Abstract

The present study aims to enrich the research on online entrepreneurship education, improve the level and ability of entrepreneurship services in China, and improve the overall success rate of entrepreneurship. Based on the technology acceptance model (TAM) proposed by researcher Davis, the study explores the application of TAM in online entrepreneurship education for new entrepreneurs. First, new entrepreneurs who are users of the online entrepreneurship education platform are selected as research objects, and then the influence of the four factors are studied, including perceived ease of use, perceived usefulness, classroom self-efficacy, and perceived external control. Finally, the hypothesis proposed is tested. Results show that online entrepreneurship education influences the perceived ease of use of the user and perceived credibility: the quality of the online entrepreneurship education has a positive impact on the user's perceived usefulness, interactivity has a positive influence on perceived ease of use and perceived usefulness, and interactivity positively influences the perceived ease of use of the user and perceived credibility. Perceived usefulness, perceived ease of use, and perceived credibility have a positive impact on the behavioral intention of the users. The proposed TAM provides some technical and theoretical support for the application of TAM in online entrepreneurship education for new entrepreneurs.

## Introduction

The development of online education is often hindered by the inconsistency of educational theories and the current technological level. Online education, as a new and innovative educational model, is easily influenced by educational concepts, the understanding of people, the external environment, and network technologies, which are impossible to be fully understood under a single network environment. Accordingly, the technology acceptance model (TAM) is introduced in online entrepreneurship education for new entrepreneurs, and its influence is specifically discussed (Liu and Yang, 2018; Qian et al., 2018).

Here, several innovation points are brought in the current research. First, the domestic research on the perception of online entrepreneurship education platforms is enriched. Entrepreneurship education is a relatively new concept, and the rapid development of online entrepreneurship education platforms has also provided a different research dimension, research of which is still in its primary stage, focusing on the qualitative analysis of the status and the problems in this field (Han, 2020; Racero et al., 2020). Under this research background, new entrepreneurs are reluctant to participate in online entrepreneurship education, so more subjective influencing factors are proposed here to analyze their influence on the use of online entrepreneurship education platforms for new entrepreneurs. As a result, the theoretical mechanism of the influence on use willingness is revealed. Second, the research on the acceptance of online entrepreneurship education platforms is explored from the perspective of new entrepreneurs (Siyal et al., 2021).

It has been argued that online entrepreneurship education can well promote entrepreneurial success by providing resources, information, and knowledge for new entrepreneurs, thereby helping them identify and seize business opportunities, which makes up for the imperfections of the entrepreneurial system, forming a more integral entrepreneurial network. Meanwhile, most recent studies on online entrepreneurship education are conducted from an overall perspective, with few studies on the entrepreneurial network. Therefore, the current research can provide a reference for the development and improvement of the online innovation and entrepreneurship industry and support for the establishment of comprehensive entrepreneurial networks. Here, TAM is introduced as a new online entrepreneurship education model, providing a fresh perspective on the study of online entrepreneurship education problems. Then, a questionnaire survey (QS) is designed based on the theory of TAM and used for quantitative analysis for the influencing factors of the new online entrepreneurship education model. The QS results show that the proposal has certain practical significance for the cultivation and sustainable development of innovation and entrepreneurship projects and incubation bases.

## Related Work

There are plenty of studies on TAM. Bhattacharyya et al. (2020) used TAM to evaluate the use of e-learning as a learning medium. Specifically, the acceptance of e-learning was evaluated as a learning medium among students majoring in Accounting and Information engineering. The samples included 60 Accounting and Information engineering students who used e-learning in the course of university. Data were collected through QS, including 30 questions, and analyzed by regression method. The test results showed that the students had a good motivation in using e-learning as a learning medium. For Accounting students, utility perception affected the motivation of students on using e-learning. Dewi and Kharisma (2020) used the model to study the factors affecting the interest of the public in watching TV sermons on the Internet. The simulation results proved the feasibility of the application of Internet TV, which had a positive impact on the actual application and the actual behaviors of the system. Accordingly, the feasibility of Internet TV broadcasting was 90.1%. Handani et al. (2020) evaluated the influence of augmented reality technology and game music on players from five evaluation indexes: the ease to use, the benefits of use, the attitude toward use, the willingness of players, and player awareness. According to the results of validation and implementation, all components in shared validation were effective and reliable. The player had a good perception of these five indexes, and the highest score was 4.10, which was involved in the ease to use and their attitude toward use. Liu et al. (2020) applied the TAM to explore the willingness of consumers and use patterns by analyzing the background information, then exploring the relationship between variables, and verifying the reliability of the measurement model using search engine marketing (SEM). The results showed that the perceived utility of users had a positive impact on the usefulness of the product, their intention to use, their curiosity, and their willingness to use. Social support also had a positive impact on the willingness of users to use a wearable device, whereas perceived curiosity had no positive effect on the willingness to use. Deng and Yuan (2020) combined TAM with social capital theory to explore the sustainable willingness of passive users. The data are collected through an online QS to test the hypotheses, and a structural equation model was used for data analysis. The findings read: trust and reciprocity played a significant and direct positive role in the persistent intention of passive users. Sharing contributed a lot to the continuous intention of passive users through trust and reciprocity. Furthermore, the usefulness and the ease to use indirectly affect the continuous willingness of passive users through shared vision, trust, and reciprocity. In summary, the application of TAM is spreading quickly (Deng et al., 2021). Based on the TAM theory, there are many achievements in the fields of online learning, systems, banking, and health. With the continuous development of the Internet, TAM is gradually evolved from enterprise systems to mobile businesses.

## TAM and Online Entrepreneurship Education for New Entrepreneurs

### Overview of TAM

The technology acceptance model is proposed in the 1980s based on social psychology theory by studying the relationship between cognitive, emotional factors, and technology application. The model is widely used in the fields of information technology. The working principle is studying the influence of technology use on the belief, attitude, and intention of users based on external observation variables. TAM is composed of four basic elements: (1) user behavior that is the actual operation behavior of the users to the new technology; (2) behavioral intention refers to the willingness of users to try new technologies; (3) perceived usefulness is the subjective understanding of users for the utility of the newly adopted technology; and (4) perceived ease of use is the degree of effort that the technology users make use of new technologies (Tambun et al., 2020).

The mathematical model of TAM is expressed as in Equations (1)–(3).


(1) $$\begin{array}{r} B=W1A+W2U \end{array}$$

(2) $$\begin{array}{r} A=W3U+W4E \end{array}$$

(3) $$\begin{array}{r} U=W5E \end{array}$$

In Equations (1)–(3), *B*, *A*, *U*, and *E* represent user behavior, behavioral intention, perceived usefulness, and perceived ease of use, respectively.

**Figure 1:**
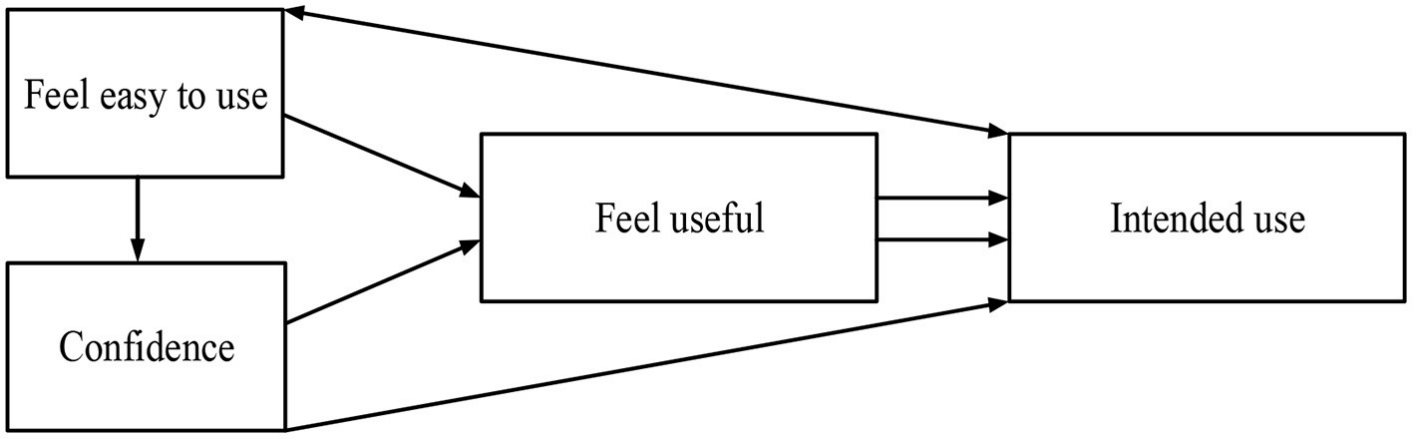
Technology acceptance model (TAM) theory.

Equation (2) shows that both perceived usefulness and perceived ease of use in the TAM model are subjective concepts, and they involve many aspects apart from information technology. Therefore, perceived usefulness and perceived ease of use determine the intention of users through their attitudes (Martasubrata and Priyadi, 2020; Syarwani and Ermansyah, 2020). Furthermore, behavioral intention is influenced both by the attitudes of users and the usefulness of the technology. TAM regards the intention of users as the most direct manifestation of user behavior. Furthermore, TAM points out that perceived usefulness is directly affected by perceived ease of use, that is, if the technology is easier to use, users can feel its usefulness (Korry, 2020; Pibriana, 2020; Ranugalih et al., 2020). In online education, the characteristics of online teaching system, teaching methods, teachers, and the difference between students are the factors that cause the understanding of the usefulness and simplicity of the technology, and then affect the attitude and intention of users toward information technology use, and ultimately, determine the actual use effect of online education (Mardhiyah et al., 2021). The TAM theory can be expressed as in Figure 1.

The TAM theory suggests that both the ease to use and the usefulness of technology affect the behavioral intention, thereby affecting the final user experience. Some researchers point out that the attitude is the response obtained through learning and experience, and the ease to use of the technology and the understanding of usefulness can be strengthened through learning, thus changing the attitudes of users (Chen, 2019; Mulyono et al., 2020; Mooya and Phiri, 2021). Therefore, the psychological factors can transmit the influence of the external environment on the new technology, and external parameters do not directly affect the use intention and behavior. The influence mainly comes from the inner understanding and attitudes of the user (Sipayung et al., 2020; Sugeng et al., 2020; Liu and Chen, 2021).

### Online Entrepreneurship Education for New Entrepreneurs

Online education is a learning model based on network technology, which is the integration of network technology with educational reform. It breaks through the rigid teaching model, widens the channel of offline teaching, and further optimizes the teaching resources (Fanuel, 2020). Online education has various forms and platforms, tailoring for people from all walks of life (Mousa et al., 2020; Musyaffi and Kayati, 2020; Wu and Song, 2020). Generally, the education model based on network technology can greatly stimulate learning interests, improve the quality of personalized education, and help cultivate comprehensive talents (Wu et al., 2020). Most significantly, innovation and entrepreneurship education can train innovative and entrepreneurial talents and improve their innovation awareness (Pelupessy and Yanuar, 2020), which is the key to innovation and entrepreneurship courses (Dixit and Prakash, 2018; Ikhsan, 2020). Meanwhile, innovation and entrepreneurship education are responsible for cultivating talents with innovative and entrepreneurial abilities. Therefore, the innovation and entrepreneurship education should be designed for diversified entrepreneurial groups and cultivate their innovative thinking and entrepreneurial ability in all stages (Sukoraharjo and Pardede, 2018; Surahmat and Tenggono, 2018; Verma et al., 2018).

### Research Methods

Here, several methods are used together, including the literature review, QS, mathematical method, and statistical analysis.

#### Literature Review

The relevant literature is read and analyzed in detail, and the results and shortcomings of previous studies are summarized to find the research entry point and theoretical support.

#### QS

Fifty new entrepreneurs in Xi'an are first selected for a trial QS, and then a formal QS is conducted from September 2020 to December 2020. Totally, 150 QSs are issued during the formal survey.

#### Statistical Analysis

SPSS was founded in 1968 with its headquarters in Chicago. The SPSS 25.0 statistical analysis software is used to verify the reliability and validity of the QS, and the corresponding structural equation model is established. Then, the structural equation model is verified, and the conclusions are obtained.

### Model Construction and QS Design

**Figure 2:**
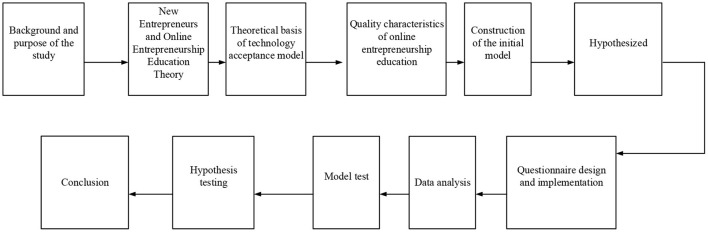
Technical roadmap.

According to the above analysis, perceived usefulness and perceived ease of use are affected by individual differences, system characteristics, social influence, and convenience (Alzubi et al., 2018; Ikram et al., 2018; Latifah et al., 2021), in which personality or demographic characteristics are individual differences, and they have a certain impact on the level of perceived usefulness and perceived ease of use (Amiruddin et al., 2021; Hong et al., 2021; Larue and Watling, 2021). Social impact refers to the degree of perception of an individual on the system under the guidance of social processes and mechanisms. Convenience refers to the support and helps for people to use the system. The technical roadmap can be expressed in Figure 2.

**Figure 3:**
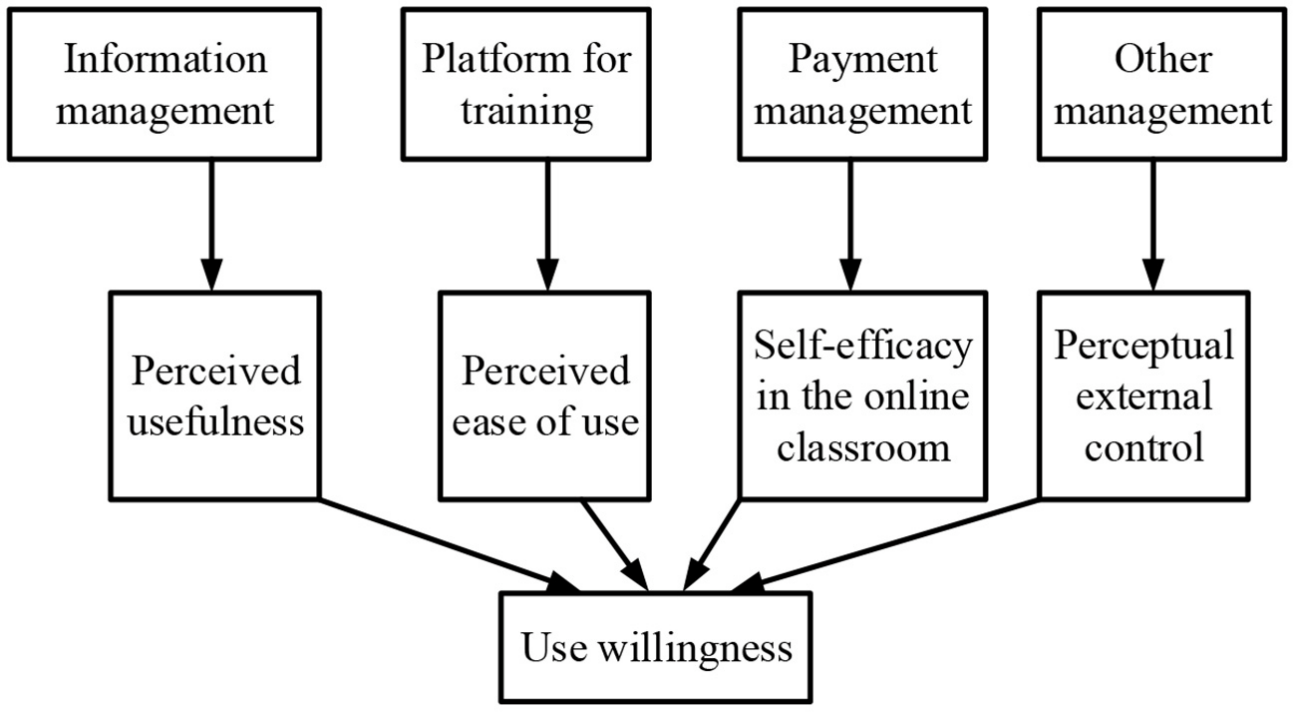
Research model of the intention of users.

Based on the theoretical basis of the TAM, the research model is proposed, as shown in Figure 3.

Based on the theoretical basis and the characteristics of online entrepreneurship education in Figure 3, the research hypotheses proposed are as follows: H1: The understanding of entrepreneurs on the usefulness of online education is positively correlated with its usefulness. H2: The understanding of entrepreneurs on the usefulness of online education is positively correlated with the attitudes of students toward the use of online education. H3: The understanding of entrepreneurs on the ease of use of online education is positively correlated with the attitudes of students toward the use of online education. H4: There is a positive correlation between the attitude and behavioral intention of an entrepreneur. H5: The understanding of entrepreneurs on the usefulness of online education is positively correlated with their behavioral intention. H6: There is a positive correlation between the behavioral intention of entrepreneurs and their behavior. After the research model and hypotheses are decided, the scale and QS are designed. Literature research is used to determine the scale, and the description of relevant researchers on the variables is referred to set the questions.

The QS is designed with three parts. The first part is to measure the characteristics of online education platforms. The second part is to measure the perception of new entrepreneurs, which includes perceived usefulness, perceived ease of use, and perceived credibility. The third part is personal information, which is put in the end to reduce the interference of personal information on subjective variables and improve the authenticity of the research (Table 1).

Table 1Questionnaire.

**Table:** 

**THE FIRST PART:**				
(1) What is the purpose of your participation in online entrepreneurship education?				
A. Access to relevant entrepreneurial knowledge	B. Obtaining relevant entrepreneurial opportunities	C. Obtaining funds	D. Access to entrepreneurial guidance	E. Other
2. What services do you want online entrepreneurship education to provide?				
A. Investment and financing services	B. Resource acquisition services	C. Advisory services in entrepreneurial domain	D. Policy release services	
3. What resources can online entrepreneurship education bring?				
A. Business	B. Customers	C. Technologies	D. Funds

**Table:** 

**THE SECOND PART:**			
**The construction of the platform**			
1. The online entrepreneurship education platform is influential and has a large number of users.			
Disagree	Be neutral	Agree	Agree totally
2. The online entrepreneurship education platform has strong operation and maintenance abilities and can operate stably.			
Disagree	Be neutral	Agree	Agree totally
3. The construction of an online entrepreneurship education platform is normative.			
Disagree	Be neutral	Agree	Agree totally
**Content**			
4. The resources obtained are rich and the content quality is good.			
Disagree	Be neutral	Agree	Agree totally
5. Entrepreneur-related activity design is valuable and attractive.			
Disagree	Be neutral	Agree	Agree totally
6. Basic services provided by the platform			
Disagree	Be neutral	Agree	Agree totally
**Interactivity**			
7. Users of the platform can interact with each other.			
Disagree	Be neutral	Agree	Agree totally
8. The platform pays attention to mental health and emotional and spiritual development.			
Disagree	Be neutral	Agree	Agree totally
**The perceived usefulness**			
9. The platform can provide a lot of useful knowledge and resources.			
Disagree	Be neutral	Agree	Agree totally
10. The platform is useful for personal growth and development.			
Disagree	Be neutral	Agree	Agree totally
**The perceived ease of use**			
11. It is easy to obtain the required resources and services.			
Disagree	Be neutral	Agree	Agree totally
12. It is easy to participate in this platform without much effort.			
Disagree	Be neutral	Agree	Agree totally
**The perceived credibility**			
13. The entrepreneurs trust each other.			
Disagree	Be neutral	Agree	Agree totally
14. Entrepreneurs trust the resources and information learned.			
Disagree	Be neutral	Agree	Agree totally

**Table:** 

**THE THIRD PART:**			
1. Your gender is ().			
Male	Female		
2. Your age is ().			
<20	>20	>40	>50
	<40	<50	
3. Your education			
A college degree	A bachelor's degree	A master's degree	A doctor's degree
4. The creation time of your venture is ().			
0–2 years	3–5 years	5–8 years	More than 8 years

The QS is conducted on 50 new entrepreneurs from July 1, 2020, to August 1, 2020, and the SPSS 25.0 statistical analysis software is used to test the reliability and validity of the data. The results of the SPSS 25.0 software analysis show that the Cronbach's alpha values of the variables are >0.8, and the Kaiser–Meyer–Olkin (KMO) test values are >0.7. This shows that the designed QS is correct and effective, and the QS can be issued on a large scale. After the correctness of the QS is verified, abundant QSs are issued immediately. From September 2020 to December 2020, QSs are issued and distributed among 150 new entrepreneurs. Totally, 150 QSs are distributed, and 145 valid QSs are recovered, with an effective recovery rate of 96.7%. Afterward, Cronbach's alpha coefficient is used to test the reliability of the QS.

**Figure 4:**
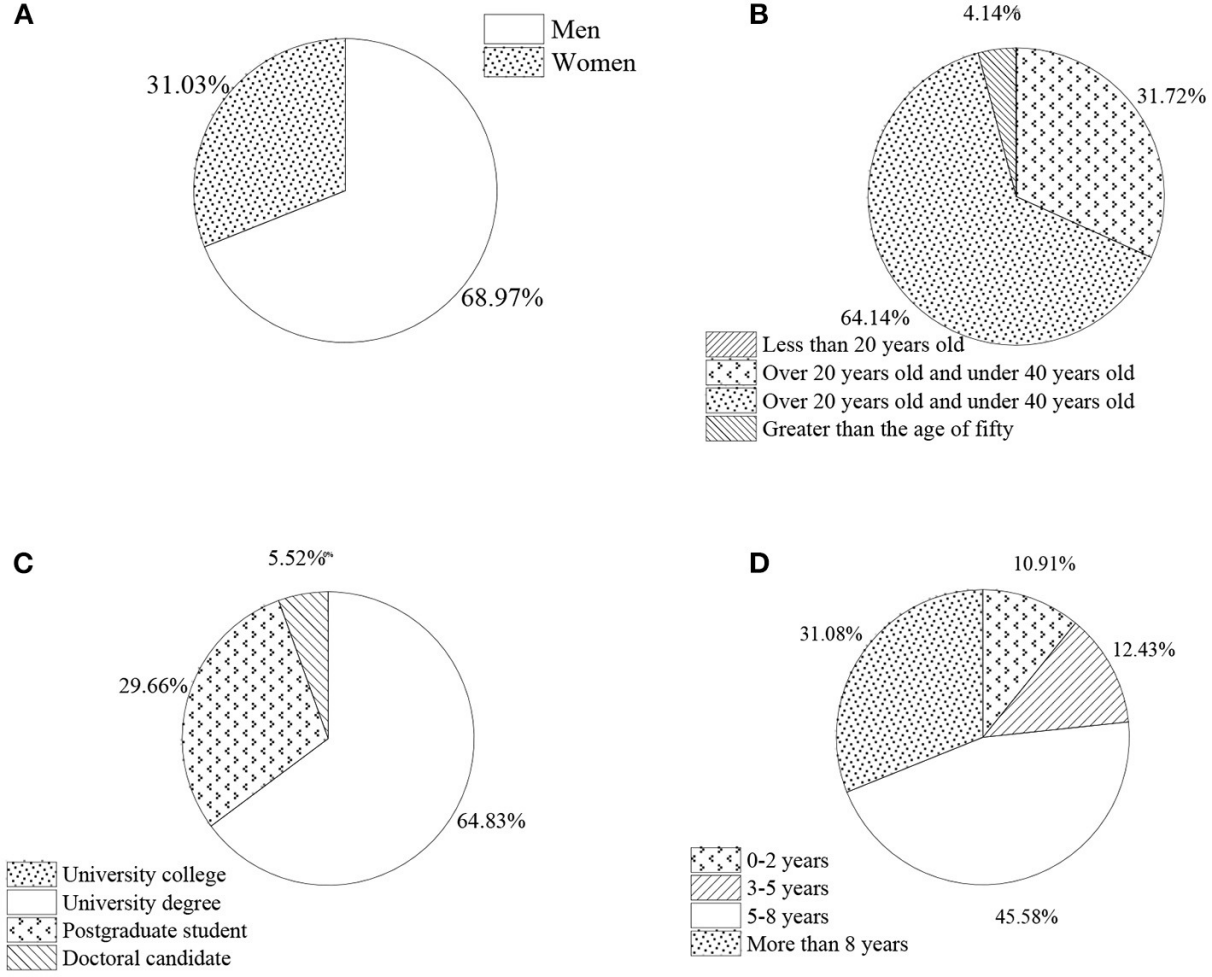
Statistical results of personal characteristics of subjects. **(A)** Gender. **(B)** Age. **(C)** Education. **(D)** Entrepreneurship period.

Figure 4 shows the statistical results of the personal characteristics of the experimental subjects.

Figure 4A shows that the proportion of men is 68.97%, and that of women is 31.03% in the QS. In terms of physical strength, men are more advantageous than women, and they are more suitable for entrepreneurship. Given the particular role of entrepreneurs, men probably are more than women. Thus, QS results are reasonable. Figure 4B indicates that the age range of subjects is from 20 to 40 years old. This shows that entrepreneurs need a certain theoretical basis for entrepreneurship, which is subject to leadership. People aged between 20 and 40 years are the most energetic groups, and they are also the main force of entrepreneurship. Figure 4C reveals that there are fewer entrepreneurs with low educational backgrounds. On the one hand, those with a low educational background often do not have the conditions to start a business; on the other hand, most entrepreneurial opportunities fall with high-tech industries, so it is difficult for entrepreneurs with a low educational background to develop in this field. Figure 4D suggests that the majority of entrepreneurs have started their businesses over 5 years on the entrepreneur service platform.

## Data test and analysis

### Analysis of QS Results

**Figure 5:**
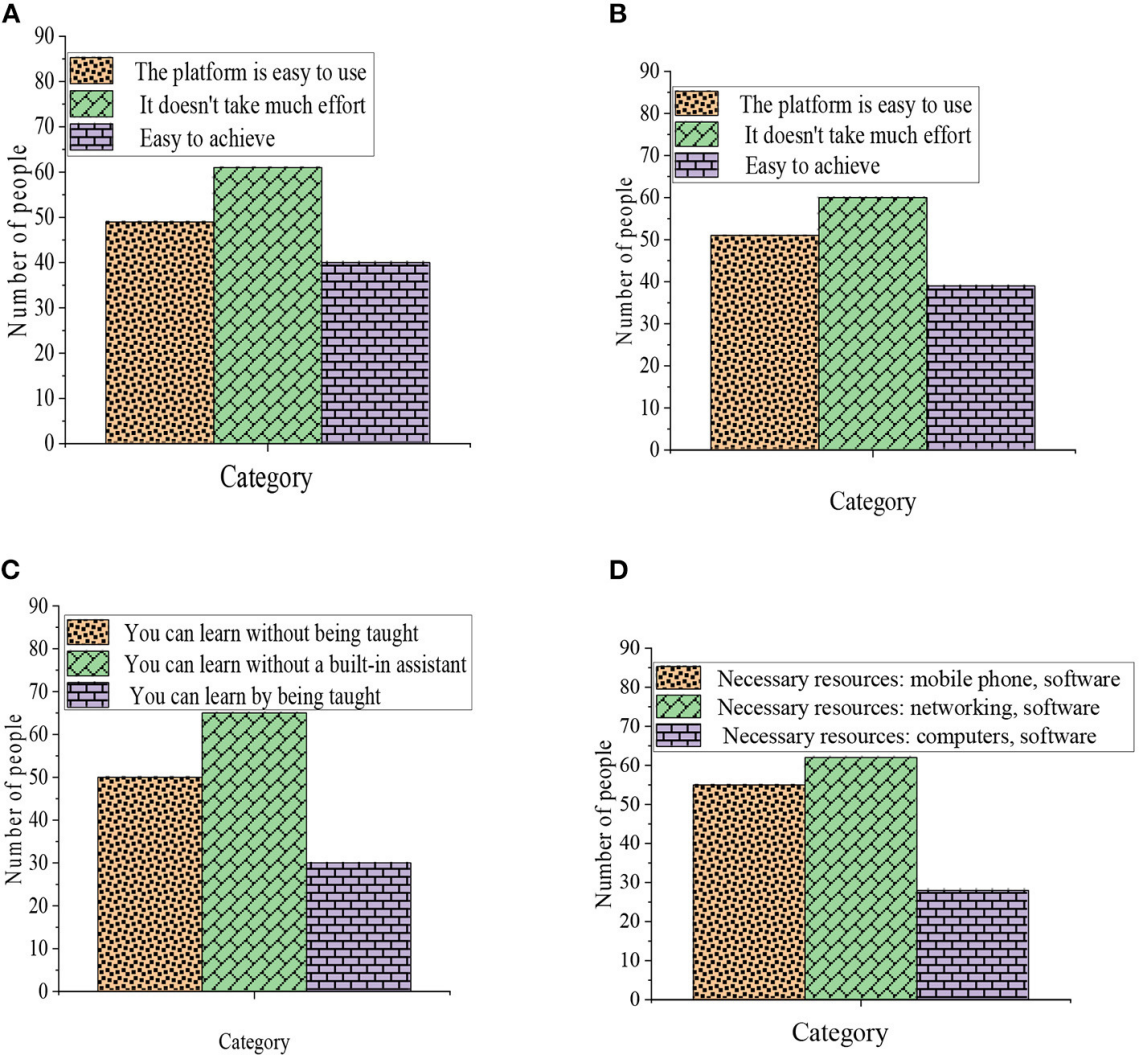
Results of questionnaire analysis. **(A)** Perceived usefulness. **(B)** Perceived ease of use. **(C)** Self-efficacy of the online classroom. **(D)** Perceived external control.

The results of the QS are shown in Figure 5.

Figure 5 indicates that the sample data conform to the normal distribution, and the number of samples is sufficient, which can be used for the establishment of a structural equation model and fitting analysis. Furthermore, the proportion of female subjects is high, but overall, the samples are representative. The data reliability and validity prove that the QS is feasible, meets the premise of structural equation model validation, and complies with the standard of data analysis and empirical test.

### Reliability and Validity of the QS

**Figure 6:**
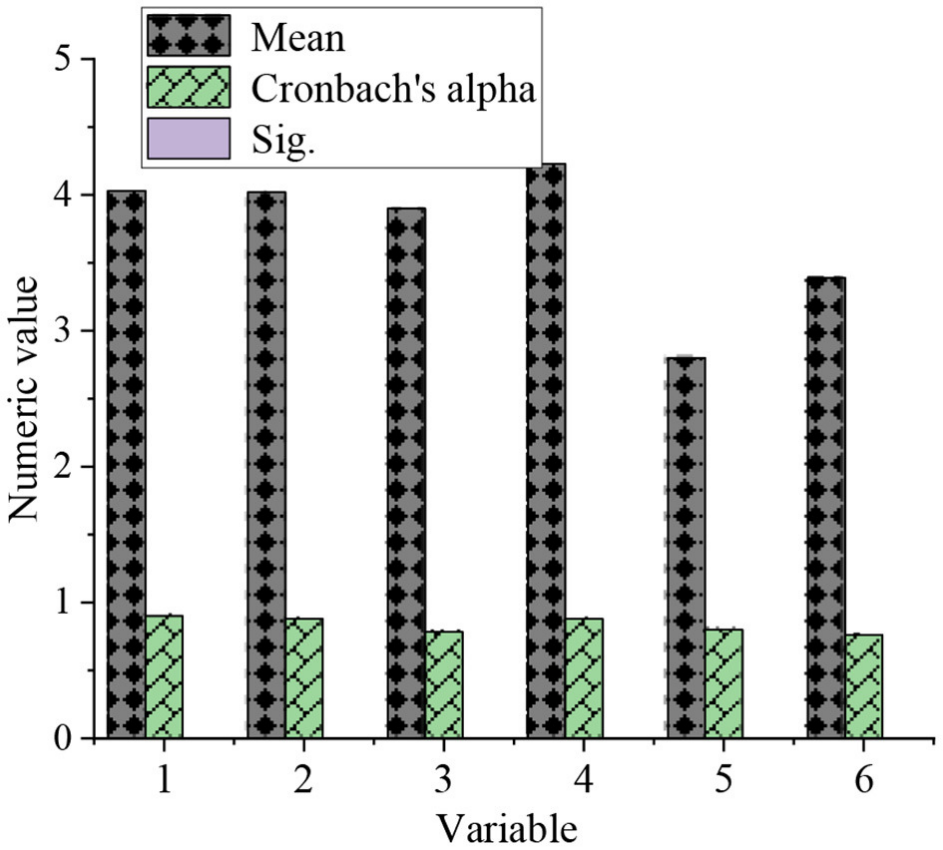
Reliability test results of the QS.

The reliability of each measurement item in the QS is tested. The Cronbach's alpha coefficient and significance index (Sig.) values of the reliability test are shown in Figure 6.

Figure 6 demonstrates that the designed QS is more reasonable and reliable, which meets the standard of reliability test. The Cronbach's alpha coefficient is 0.922, and the Sig. value is 0.000. The test results show that the internal consistency, reliability, and repeatability of internal measurement items are good. The QS is reliable.

**Figure 7:**
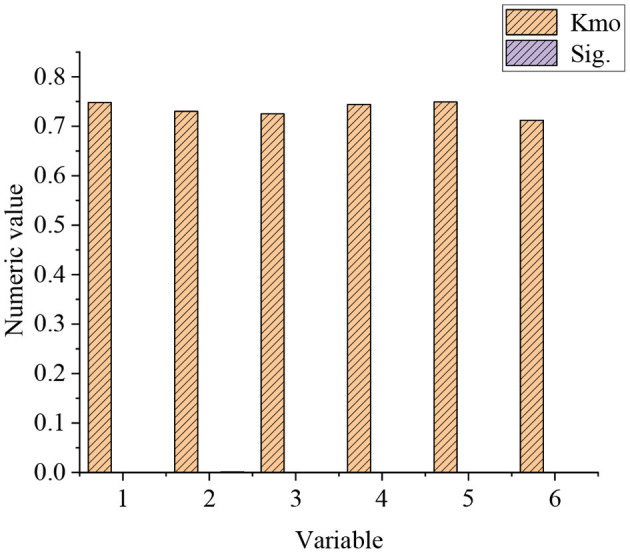
KMO test of the whole QS.

The KMO sample is used to test whether the sample can carry out factor analysis. The KMO values of each variable in the QS and the statistical results of the Bartlett sphere test are shown in Figure 7.

The KMO values of perceived ease of use, perceived usefulness, online classroom self-efficacy, and perceived external control are 0.748, 0.730, 0.725, and 0.744, respectively. The Chi-square values of perceived ease of use are 536.213, perceived usefulness is 462.512, self-efficacy is 289.112, and perceived external control is 483.112. Overall, the KMO test values of each variable in the QS are >0.7, all Sig. values are 0, and the chi-square of the spherical test is >200. This shows that the variables in the QS are suitable for the factor analysis.

### Factor Analysis Results

**Figure 8:**
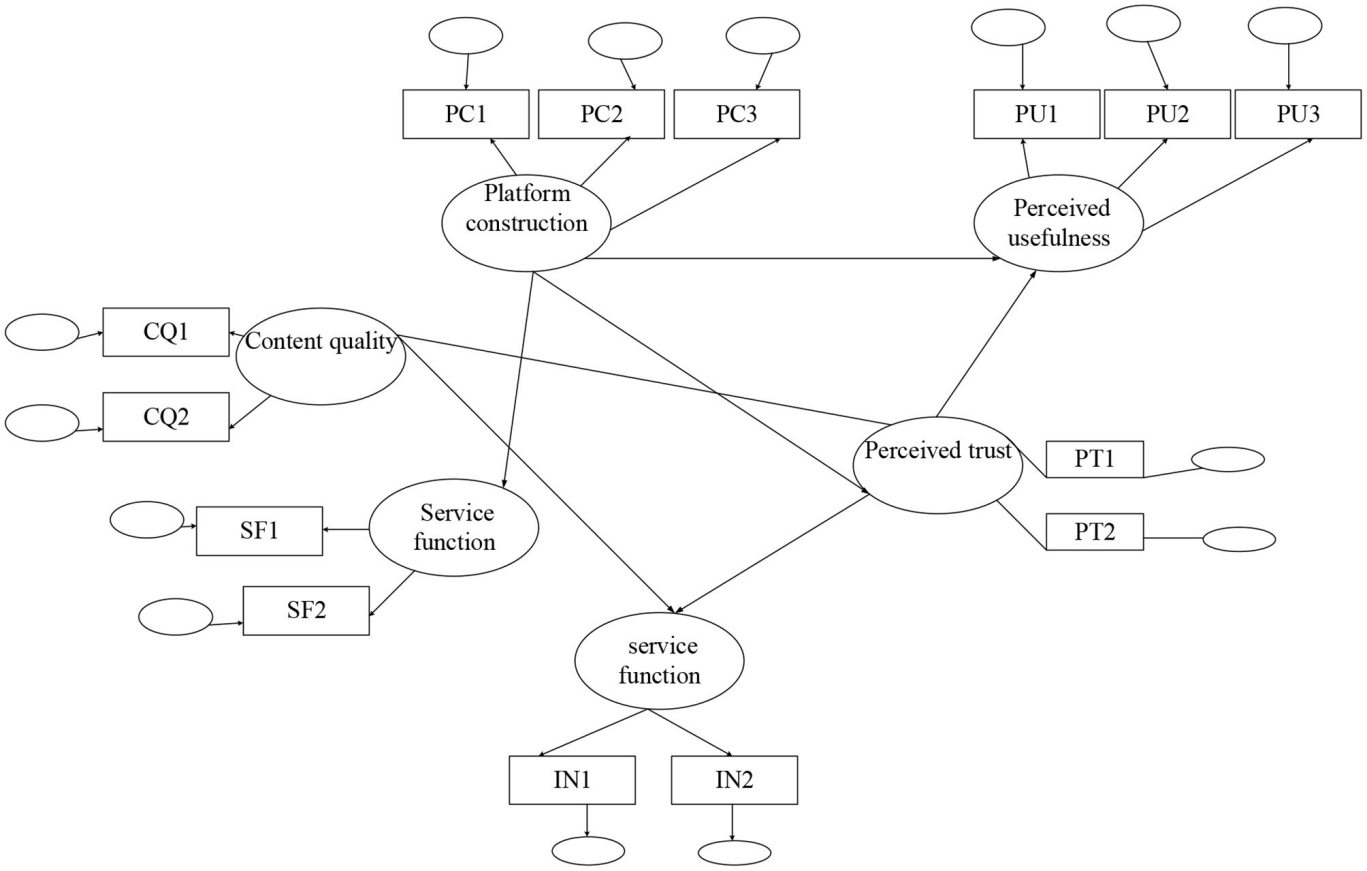
The structure equation of the model.

The results of Analysis of Moment Structures (AMOS) modeling are shown in Figure 8.

**Figure 9:**
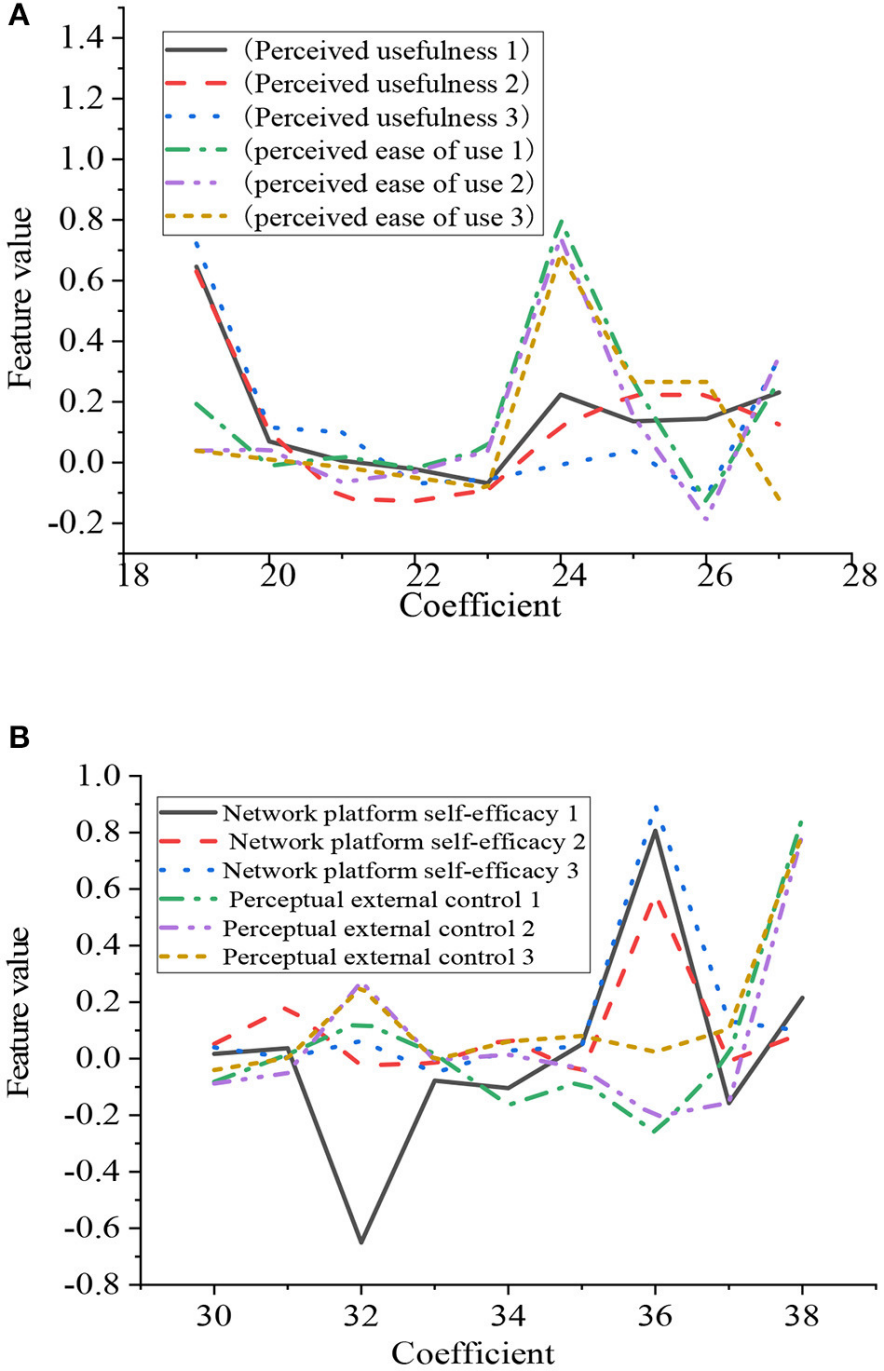
Factor analysis. **(A)** Perceived usefulness and perceived ease of use. **(B)** Self-efficacy, perception, and external control of the online classroom.

A better structure validity indicates that there is a high correlation between different measures under the same construction, whereas a better discriminant validity means that there is a low correlation between measures under different constructions. The quality of aggregate validity and discriminant validity affects the accuracy of the QS. Therefore, the data analysis software is used to extract the principal analysis factors in the data, measure the individual factor load, and extract multiple factors. The factor analysis results are shown in Figure 9.

Figure 9 shows that the observed values of the same variable in the observation scale have the highest load on the same factor, and the observed variables are closely related to the load. The highest load of the three observation variables of perceived usefulness is in the first factor. The highest load of the three observation variables of perceived ease of use is in the sixth factor. The highest load of the three observation variables of self-efficacy is in the seventh factor. The highest load of the three observation variables of perceived external control is in the ninth factor. All the highest factor loads exceed 0.7. The perceived credibility has no significant direct impact on perceived usefulness, whereas it has no significant direct impact on the perceived ease of use. In short, the results of the analysis are consistent with the hypotheses. The proposed model is correct and effective.

According to the hypotheses, and the test results of the reliability and validity of the QS samples, the structural equation model is constructed in the AMOS software. Amos 22.0 is a part of IBM SPSS 22 series. It is a professional graphical modeling tool created by IBM, IBM, the international business machine company, founded in the United States in 1914. Three regression models are used to test five hypotheses. The first regression model tests hypothesis H1: the independent variable is perceived ease of use, and the dependent variable is perceived usefulness. The second regression model verifies H2 and H3: the independent variables are perceived ease of use and perceived usefulness, and the dependent variable is the attitude toward the use. The third proves H4 and H5: perceived usefulness and the attitude toward use are independent variables, and use intention is the dependent variable. The model test results are as follows:

1. In the first test model of hypothesis H1, the regression equation of the relationship between perceived ease of use and perceived usefulness has a small decision coefficient. The values are *F* = 23.11 and *p* < 0.005, which indicates that H1 is proved.
2. In the test of H2 and H3, the coefficient of regression equation about the relationship between perceived ease of use and attitude toward use in the H2 hypothesis is little. The results of the test are *F* = 120.11 and *p* < 0.005. The variance inflation factor (VIF) = 2.103 is far less than the threshold, which implies that the equation does not have multicollinearity. In the H3 hypothesis, the coefficient of regression equation between perceived usefulness and attitude toward use is little, but *F* = 51.22, *p* < 0.01, and VIF = 2.14 show that H2 and H3 are supported.
3. In the test of H4, H5, and H6, the results show that perceived usefulness is positively correlated with behavioral intention, and the attitude toward use is positively correlated with behavioral intention. According to the statistical data, H4 and H5 are confirmed.

## Discussion and Suggestions

Two conclusions are drawn:

1. Ease of use and usefulness are two important factors that affect the acceptance and use of online entrepreneurship education platforms by new entrepreneurs. Although the average age of new entrepreneurs decreases and the application ability of computers is improved, its ease of use is still a decisive factor for new entrepreneurs to accept and use teaching software.
2. Usefulness and ease of use, and the attitude toward online entrepreneurship education platforms and behavioral intention of new entrepreneurs, should become important reference factors in the evaluation and selection of teaching software. How to know and master the views of new entrepreneurs on an online entrepreneurship education platform becomes an essential job that the Research and Development (R&D) department of an online entrepreneurship education platform needs to pay attention to.

## Conclusions

In this study, the use intention model of new entrepreneurs is implemented in online innovation and entrepreneurship education classrooms based on the TAM theoretical framework. The model takes perceived usefulness, perceived ease of use, classroom self-efficacy, and perceived external control as the intermediate variables, and uses intention as the dependent variable. The corresponding hypotheses are put forward, and the influence mechanism of the intention of new entrepreneurs for online innovation and entrepreneurship education is revealed. The results prove the validity of the proposed hypotheses. The external variables on the platform will have different degrees of influence on the user perception. The construction of the online education platform will have a direct impact on the perceived ease of use and perceived credibility of users. The quality of online education courses will have a direct impact on the perceived usefulness. Interactivity has a certain influence on perceived ease of use and perceived usefulness, and there is a mutual influence between user perceptions (perceived ease of use and perceived usefulness).

There are three drawbacks: first, the original TAM is developed for the corporate computer software system, which is adjusted here for the online entrepreneurship education curriculum platform, and the influence factor of use intention is not fully considered. The second point is the limitation of the QS samples. Most recruited new entrepreneurs are concentrated in Shaanxi, this regional limitation may have a certain influence on the research conclusion. The third point is that the mutual relationship among various factors has not been fully verified. The structural equation model is used to verify the validity and reliability of the theoretical model. The hypothesized path relationship has not presented a detailed description of the potential path relationships of various variables. In the future, the application orientation of the proposed model is to provide customized services for new entrepreneurs based on the basic information analysis of users, user behavior statistics, and big data analysis technology, and meet more personalized user needs. Personalization of service can provide the correct service content based on the user needs and the right service direction. Thus, according to the needs of entrepreneurs, “one-to-one” guidance can be adopted to provide entrepreneurs with consultation and planned services.

## Data Availability Statement

The original contributions presented in the study are included in the article/supplementary material, further inquiries can be directed to the corresponding author/s.

## Ethics Statement

The studies involving human participants were reviewed and approved by The Education University of Hong Kong Ethics Committee. The patients/participants provided their written informed consent to participate in this study. Written informed consent was obtained from the individual(s) for the publication of any potentially identifiable images or data included in this article.

## Author Contributions

All authors listed have made a substantial, direct and intellectual contribution to the work, and approved it for publication. YS: writing—original draft, conceptualization, methodology, and software. ML: writing—original draft, reviewing, and supervision.

## Funding

This study was supported by Guangdong Provincial Education Science 13th Five-Year Plan 2020 Annual Research Project Research on the Collaborative Development Platform of Vocational Education Network Curriculum in Guangdong-Hong Kong-Macao Greater Bay Area (No. 2020GXJK321).

## Conflict of Interest

The authors declare that the research was conducted in the absence of any commercial or financial relationships that could be construed as a potential conflict of interest.

## Publisher's Note

All claims expressed in this article are solely those of the authors and do not necessarily represent those of their affiliated organizations, or those of the publisher, the editors and the reviewers. Any product that may be evaluated in this article, or claim that may be made by its manufacturer, is not guaranteed or endorsed by the publisher.
